# Suicide rate and social environment characteristics in South Korea: the roles of socioeconomic, demographic, urbanicity, general health behaviors, and other environmental factors on suicide rate

**DOI:** 10.1186/s12889-022-12843-4

**Published:** 2022-03-01

**Authors:** Hyemin Jang, Whanhee Lee, Yong-ook Kim, Ho Kim

**Affiliations:** 1 Department of Statistics, Ewha Womans University, Seoul, Korea; 2 School of the Environment, Yale University, New Haven, CT USA; 3 Population Research Lab, Department of Public Health Science, Graduate School of Public Health, Seoul National University, Seoul, Korea; 4 Department of Public Health Science, Graduate School of Public Health, & Institute of Health and Environment, Seoul National University, 1 Gwanak-ro, Gwanak-gu, Seoul, 151-742 Republic of Korea

**Keywords:** Suicide, Social environment factors, Longitudinal study, Bayesian hierarchical model

## Abstract

**Background:**

Suicide is a serious worldwide public health concern, and South Korea has shown the highest suicide rate among Organisation for Economic Co-operation and Development (OECD) countries since 2003. Nevertheless, most previous Korean studies on suicide had limitations in investigating various social environment factors using long-term nationwide data. Thus, this study examined how various social environment characteristics are related to the suicide rate at the district-level, using nationwide longitudinal data over 11 years.

**Methods:**

We used the district-level age-standardized suicide rate and a total of 12 annual social environment characteristics that represented socioeconomic, demographic, urbanicity, general health behaviors, and other environmental characteristics from 229 administrative districts in South Korea. A Bayesian hierarchical model with integrated Laplace approximations (INLA) was used to examine the spatiotemporal association between the rate of suicide and the social environment indicators selected for the study.

**Results:**

In the total population, the indicators “% of population aged 65 and older eligible for the basic pension”, “% vacant houses in the area”, “% divorce”, “% single elderly households”, “% detached houses”, “% current smokers”, and “% of population with obesity” showed positive associations with the suicide rate. In contrast, “% of people who regularly participated in religious activities” showed negative associations with suicide rate. The associations between these social environment characteristics and suicide rate were generally more statistically significant in males and more urbanized areas, than in females and less urbanized areas; however, associations differed amongst age groups, depending on the social environment characteristic variable under study.

**Conclusions:**

This study investigated the complex role of social environments on suicide rate in South Korea and revealed that higher suicide rates were associated with lower values of socioeconomic status, physical exercise, and religious activities, and with higher social isolation and smoking practice. Our results can be used in the development of targeted suicide prevention policies.

**Supplementary Information:**

The online version contains supplementary material available at 10.1186/s12889-022-12843-4.

## Background

The World Health Organization (WHO) reported that more than 700,000 people die by suicide every year, and accounted for 1.3% of all deaths in 2019 [1]. In an effort to reduce suicidal deaths globally, the WHO Mental Health Action Plan 2013–2020 has been extended to 2030. Among Organization for Economic Cooperation and Development (OECD) countries, South Korea (hereafter referred to as Korea) has had the highest suicide rate for the period 2003 to 2019 (24.6 per 100,000 persons; Statistics Korea, 2019). Suicide is the fifth leading cause of death in Korea, and this trend has been more pronounced in the younger population aged 10–59 years (suicide is the first and second leading cause of death in people aged 10–39 years and aged 40–59 years, respectively) than in the older population aged 60 years and older (Statistics Korea, 2019). The current statistics prompts us to recognize suicide as a public health priority, and it has become more important to examine various factors that may contribute towards suicide.

Since Durkheim’s initial sociological research regarding the effects of social factors on suicide [2], a number of studies have consistently reported that cultural/social factors, such as social integration, socioeconomic status, and residential conditions, are closely related to suicide [3]. However, many Korean studies in the previous decade have examined the temporal trend in the suicide rate and individual-level risk factors [4, 5], and few studies have investigated the roles of social environmental characteristics on the suicide rate. Cheong et al. showed that urbanicity was associated with regional suicide (that is, rural areas showed higher suicide rates compared with urban areas), and regional economic status was also related to suicide rate in the elderly population [6]. Kim also reported that the regional income-levels, prevalence of heavy drinking, and the % elderly (people aged 65 years and older) in the community were associated with suicide rates [7]. However, most previous studies in Korea are limited in their examination of various social environment factors and/or have used suicide data from relatively short study periods (less than 5 years).

Therefore, to address these knowledge gaps, this study examined the complex roles of social environment characteristics, including multiple regional socioeconomic, demographic, urbanicity, general health behaviors, and other environmental factors on suicide rate, using district-level nationwide longitudinal data from 2008 through 2018, from 229 administrative districts in Korea. In addition, we investigated the different effects of social environment characteristics on the suicide rate in areas grouped according to their urbanization level, for male and female sexes, and for different age groups.

## Methods

### Suicide data

We obtained population-based longitudinal data on annual mortality counts for each district for the period 2008–2018 from the Korea National Statistics Office. The mortality data include the location of death (that is, the administrative district), and the age and sex of the deceased. Data for our study were extracted from the national mortality data, which used the 10^th^ revision of the International Classification of Diseases (ICD-10) codes to define deaths due to suicide (designated in the range X60–X84).

In this study, a crude suicide rate was calculated for each district in each study year based on the entire population living in Korea during the study period. We also calculated the age-standardized suicide rate per 100,000 persons for each study year (hereafter, suicide rate), using the 2005 resident-registered population in Korea [8]. The age-standardized suicide rate for each study year was used in the statistical analyses.

### Social environment characteristics

Based on previous studies that identified the association between suicide and socioeconomic status, social isolation, social activities, and health status [1, 3, 6, 7, 9–11], this study collected a total of 12 district-level annual indicators from all 229 administrative districts in Korea (that is, “si/gun/gu”, which are second-level basic local administrative districts; the median population of all districts was 143,461 during the study period) to address the complex effects of social environment characteristics on suicide rate. These indicators can be broadly categorized into: 1) Socioeconomic status (% of population aged 65 and older eligible for the basic pension, % vacant houses in the area), 2) Isolation (% divorce, % single elderly households, % detached houses), 3) Recreational opportunities / religious and physical activities (% of people who regularly participated in religious activities, number of sports facilities per 1,000 persons, park area per person (km^2^) and 4) Health behavior characteristics (% current smokers, % of people exhibiting high risk drinking, % of population with recognized stress, % of population with obesity). These indicators were obtained from the database of community health outcomes and health determinants that is maintained by the Korean Centers for Disease Control and Prevention [8]. If variables were missing for a year, we linearly interpolated or extrapolated their values using available data [12]. Detailed information on these indicators is provided in the Supplementary Materials (A. Information on Social Environment Indicators and Table S1).

### Sub-group analysis

Korea is one of the world’s most urbanized countries (87.5% urban population in 2017) and experienced rapid urbanization during the 1960–90 s. Thus, we postulated social environment characteristics may be closely related to urbanization level. To examine the effects of the social environment characteristics on suicide rate according to the urbanization level of areas, we divided 229 districts into three groups using population density (person per km^2^, an indicator of urbanization level). Seventy six districts with average population densities in the range 19.8–136.2 persons per km^2^ (corresponding to the 0^th^–33.3^th^ percentiles) were categorized as low-density areas, 77 districts with average population densities in the range 136.8–2077.1 persons per km^2^ (corresponding to the 33.3^th^–66.7^th^ percentiles) were regarded as mid-density areas, and the remaining 77 districts with population densities in the range 2811.0–28,081.1 persons per km^2^ (corresponding to the 66.7^th^–100^th^ percentiles) were classified as high-density areas.

In addition, we used the age-standardized suicide rates for each study year, which were calculated for groups of people aged 10–39 years, 40–59 years, and 60 years and older) in each district. Suicide rates were calculated for each sex (male and female) per district.

### Statistical analysis

We applied a Bayesian hierarchical model to examine the spatiotemporal association between the rate of suicide and time-varying annual social environment characteristics, using integrated nested Laplace approximations (INLA) [13]. All 12 time-varying annual social environment characteristics were included as linear terms in a Bayesian hierarchical model. To address temporal and spatial confounding, we fitted the model with district-specific random intercepts and adjusted for indicator variables for the years 2008–2018 and the coordinate (longitude and latitude) of each district. In addition, a random intercept using the Besag-York-Mollie (BYM) method [14] was considered in the model to address residual spatial correlations among districts.

From this model, we estimated the association between suicide rate and each social environment characteristic as the change in suicide rate per unit change in each social environment characteristic variable. All analytical procedures were repeated for each sub-population (low-, mid-, and high-density areas; male and female sexes; and age groups). The strength of the suicide-social environment relationship among sub-populations was interpreted based on the number of social environment indicators that showed a statistically significant association with suicide rates (i.e., *p*-value < 0.05). For all statistical analyses, we used R statistical software (version 4.0.3). For the sensitivity analysis, we repeated the main analysis excluding the social environment indicators that showed high correlations (Pearson’s correlation > 0.6) with other variables.

## Results

**Fig. 1:**
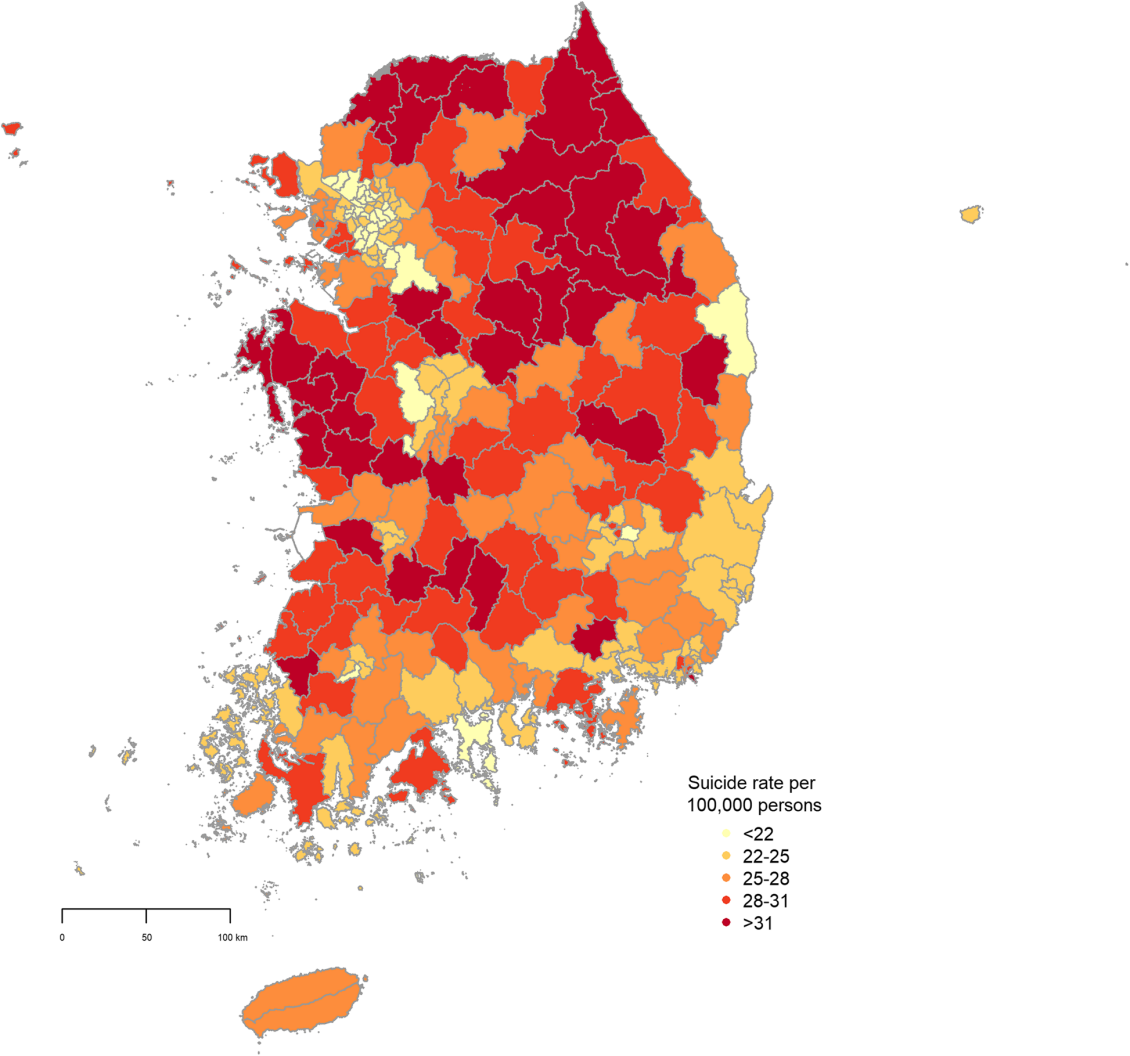
Geographical distribution of average suicide rates across 229 districts in Korea during the period 2008–2018

Table 1 displays descriptive statistics of suicide rates and confounders (median population of all districts: 143,461). During the entire study period, low-density areas, males, and people aged 60 years and older showed higher suicide rates on average, compared to mid- or high-density areas, females, and people aged 40–59 or 10–39 years. Low-density areas showed the highest suicide rate across all sub-populations (sexes and age groups), except for people aged 60 years and older. The geographical distribution of average suicide rates for the period 2008–2018 across 229 districts can be seen in Fig. 1. The spatial distribution of the average of each social environment characteristic is displayed in Figure S1 and the correlations among these variables are reported in Table S2. We found that the correlations among these 12 variables were not sufficiently large (Pearson’s correlations < 0.4), except for a few variables. For instance, ‘% of population aged 65 and older eligible for the basic pension’ and ‘% detached houses’ (Pearson’s correlations > 0.6).

**Table 1:** Descriptive statistics of suicide rates and social environment characteristics in the total population and at different urbanization levels

	**Total**	**Low-density areas**	**Mid-density areas**	**High-density areas**
Total suicide rate	27.07 (18.30, 37.86)	29.74 (17.50, 44.10)	27.43 (19.21, 36.40)	24.04 (18.15, 30.60)
Male suicide rate	38.59 (25.00, 55.10)	42.64 (23.14, 64.30)	39.31 (27.70, 54.29)	33.83 (24.70, 43.90)
Female suicide rate	16.21 (7.59, 25.90)	16.95 (3.70, 32.71)	16.40 (9.10, 24.90)	15.29 (9.80, 21.15)
Aged 10 ~ 39y suicide rate	20.17 (9.92, 32.17)	22.17 (5.29, 41.74)	20.01 (11.50, 29.85)	18.34 (12.29, 24.59)
Aged 40 ~ 59y suicide rate	36.84 (22.08, 54.90)	41.70 (19.40, 66.34)	36.97 (23.83, 51.23)	31.86 (22.40, 42.28)
Aged 60 + y suicide rate	56.89 (30.89, 88.85)	59.32 (26.75, 99.55)	60.75 (35.21, 93.33)	50.62 (32.10, 71.99)
% of population aged 65 and older eligible for the basic pension	70.50 (56.94, 84.46)	80.65 (72.31, 86.54)	68.65 (59.74, 78.45)	62.21 (48.24, 73.73)
% vacant houses in the area	8.49 (2.68, 14.55)	12.57 (8.80, 16.51)	8.77 (4.54, 13.60)	4.14 (1.90, 7.24)
% divorce	2.19 (1.70, 2.70)	2.07 (1.70, 2.50)	2.33 (1.90, 2.80)	2.17 (1.70, 2.70)
% single elderly households	21.22 (14.94, 28.48)	25.84 (20.10, 30.97)	20.24 (14.07, 25.11)	17.59 (14.10, 21.97)
% detached houses	44.33 (10.10, 87.15)	77.43 (61.54, 90.47)	36.28 (14.42, 62.84)	19.28 (6.33, 35.26)
% of people who regularly participated in religious activities	25.15 (16.55, 34.10)	22.36 (14.10, 30.60)	25.33 (16.70, 34.00)	27.76 (20.15, 35.08)
Number of sports facilities per 1,000 persons	0.05 (0.00, 0.11)	0.09 (0.02, 0.19)	0.04 (0.01, 0.07)	0.01 (0.00, 0.01)
Park area per person (km^2^)	22.66 (2.73, 41.42)	26.47 (9.68, 49.65)	31.09 (8.27, 60.37)	10.47 (0.62, 22.82)
% current smokers	23.89 (19.96, 27.84)	24.05 (20.40, 27.80)	24.13 (19.94, 28.20)	23.49 (19.70, 27.45)
% of people exhibiting high-risk drinking	17.91 (12.60, 22.90)	17.93 (11.40, 24.30)	18.24 (13.14, 23.00)	17.57 (13.50, 21.30)
% of population with recognized stress	27.00 (20.66, 32.60)	24.36 (18.20, 30.50)	27.68 (22.54, 32.50)	28.97 (24.30, 33.40)
% of population with obesity	26.52 (20.60, 34.74)	26.95 (20.35, 35.70)	27.14 (21.20, 35.26)	25.46 (20.30, 33.00)

*Note*) Numbers: Average (10th percentile, 90th percentile). Seventy six districts with average population densities in the range 19.8–136.2 persons per km^2^ (corresponding to the 0th–33.3th percentiles) were categorized as low-density areas, 77 districts with average population densities in the range 136.8–2077.1 persons per km^2^ (corresponding to the 33.3th –66.7th percentiles) were regarded as mid-density areas, and the remaining 77 districts with population densities in the range 2811.0–28,081.1 persons per km^2^ (corresponding to the 66.7th –100th percentiles) were classified as high-density areas

**Fig. 2:**
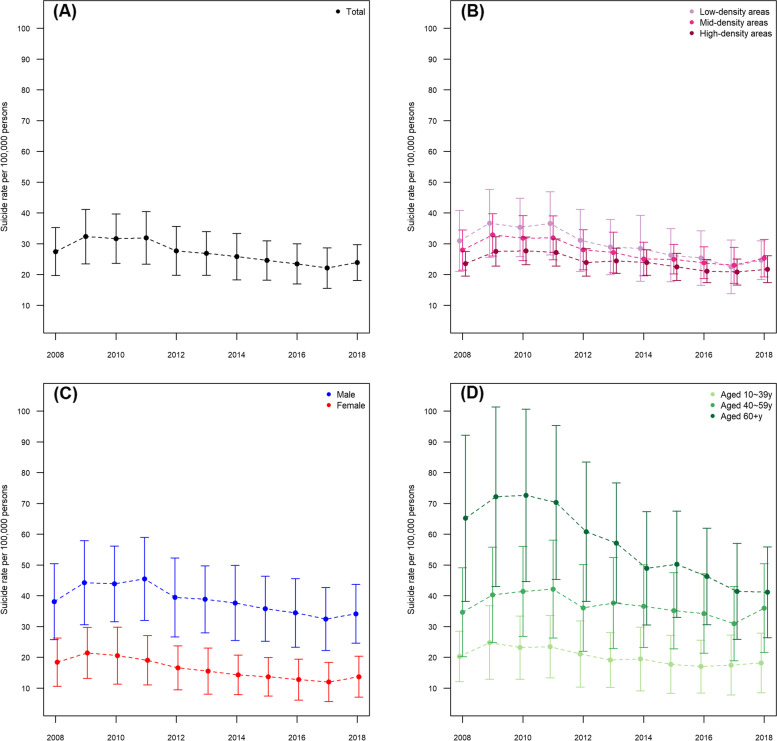
Annual trends in the suicide rate in the total population and for each sub-population: (**A**) Total population, (**B**) Areas divided by population density, (**C**) Sex, and (**D**) Age groups

Figure 2 shows the annual trend in the suicide rate in the total population and for each sub-population. In the total population, the suicide rate gradually decreased from 2009, except for an increase in 2018. We presume this is the result of the government’s active suicide prevention efforts, starting with the Suicide Prevention Law which was enacted in 2011. This decreasing pattern was most prominent in low-density areas, and among males and people aged 60 years and older.

Table 2 shows the association between social environment characteristics and suicide rate in the total population and for low-, mid-, and high-density areas. In the total population, higher values of the indicators, “% of population aged 65 and older eligible for the basic pension”, “% vacant houses in the area”, “% divorce”, “% detached houses”, and “% current smokers” were associated with higher suicide rates. In contrast, “% of people who regularly participated in religious activities” showed a negative association with suicide rate in the total population. In general, the associations observed with these social environment characteristics were more statistically significant (i.e. lower *p*-values) in high- and mid-density areas than in low-density areas.

**Table 2:** Association between social environment characteristics and suicide rate in the total population and at different urbanization levels

	**Total**	**Low-density areas**	**Mid-density areas**	**High-density areas**
% of population aged 65 and older eligible for the basic pension	0.09 (0.04, 0.14)^*^	0.08 (-0.10, 0.26)	0.08 (0.02, 0.14)^*^	0.09 (0.03, 0.14)^*^
% vacant houses in the area	0.16 (0.07, 0.25)^*^	0.34 (0.13, 0.54)^*^	0.03 (-0.09, 0.15)	0.20 (0.04, 0.35)^*^
% divorce	1.88 (0.99, 2.76)^*^	-2.23 (-4.47, 0.01)	3.81 (2.70, 4.92)^*^	4.13 (2.98, 5.28)^*^
% single elderly households	0.05 (-0.02, 0.12)	-0.32 (-0.66, 0.02)	0.06 (0.00, 0.12)^*^	0.13 (-0.08, 0.34)
% detached houses	0.04 (0.02, 0.06)^*^	0.03 (-0.04, 0.11)	0.09 (0.06, 0.11)^*^	-0.01 (-0.05, 0.03)
% of people who regularly participated in religious activities	-0.11 (-0.17, -0.05)^*^	-0.05 (-0.17, 0.07)	-0.25 (-0.32, -0.18)^*^	0.02 (-0.06, 0.10)
Number of sports facilities per 1,000 persons	-2.85 (-6.23, 0.52)	-3.66 (-8.59, 1.26)	-2.60 (-13.87, 8.67)	-35.12 (-68.55, -1.74)^*^
Park area per person (km^2^)	-0.01 (-0.02, 0.00)	0.04 (-0.01, 0.08)	-0.01 (-0.02, 0.00)	-0.01 (-0.03, 0.00)^*^
% current smokers	0.17 (0.06, 0.28)^*^	0.20 (-0.06, 0.47)	0.07 (-0.09, 0.22)	0.11 (0.00, 0.22)^*^
% of people exhibiting high-risk drinking	-0.05 (-0.13, 0.03)	-0.02 (-0.19, 0.14)	-0.05 (-0.16, 0.06)	-0.01 (-0.09, 0.08)
% of population with recognized stress	-0.01 (-0.07, 0.06)	0.02 (-0.12, 0.16)	0.02 (-0.08, 0.11)	0.04 (-0.02, 0.11)
% of population with obesity	0.09 (0.00, 0.18)	0.01 (-0.20, 0.21)	0.01 (-0.13, 0.15)	0.02 (-0.09, 0.12)

*Note*) Numbers: Changes in suicide rate per 100,000 persons (95% credible interval). Seventy six districts with average population densities in the range 19.8–136.2 persons per km^2^ (corresponding to the 0th–33.3th percentiles) were categorized as low-density areas, 77 districts with average population densities in the range 136.8–2077.1 persons per km^2^ (corresponding to the 33.3th –66.7th percentiles) were regarded as mid-density areas, and the remaining 77 districts with population densities in the range 2811.0–28,081.1 persons per km^2^ (corresponding to the 66.7th –100th percentiles) were classified as high-density areas

^*^p < 0.05

Table 3 shows the sex-specific and age group-specific associations between social environment characteristics and suicide rate. The associations with social environment characteristics observed in the total population were generally more significant in males than in females, and a negative association between suicide rate and “% of people who regularly participated in religious activities” was observed in males. Meanwhile, age groups showed heterogeneous associations of social environment characteristics with suicide risk. First, “% single elderly households” and “% detached houses” were positively associated with suicide rate only for people aged 10–39 and 40–59 years. Moreover, the negative relationship between suicide rate and “% of people who regularly participated in religious activities” was more prominent in people aged 40–59 years and 60 years and older, than in people aged 10–39 years. Similarly, the negative association of suicide rate with “park area per person (km^2^)” and “number of sports facilities per 1,000 persons” were observed only in people aged 10–39 years. In contrast, positive associations of suicide rate with “% vacant houses in the area”, “% divorce”, “% current smokers”, and “% population with recognized stress” were observed across all age groups. Finally, the sensitivity analysis (Table S3) revealed that our main results are robust to the inclusion/exclusion of variables that were highly correlated with other social environment indicators, based on the directionality and statistical significance of the associations of the variables with suicide rates.

**Table 3:** Sex-specific and age group-specific associations between social environment characteristics and suicide rate

	**Male**	**Female**	**Aged 10 ~ 39y**	**Aged 40 ~ 59y**	**Aged 60 + y**
% of population aged 65 and older eligible for the basic pension	0.16 (0.09, 0.24)^*^	0.03 (-0.02, 0.07)	0.02 (-0.03, 0.08)	0.06 (-0.02, 0.14)	0.35 (0.23, 0.46)^*^
% vacant houses in the area	0.21 (0.06, 0.36)^*^	0.09 (0.00, 0.18)	0.20 (0.08, 0.32)^*^	0.23 (0.06, 0.40)^*^	0.53 (0.27, 0.79)^*^
% divorce	2.96 (1.57, 4.34)^*^	1.10 (0.24, 1.96)^*^	1.47 (0.35, 2.59)^*^	4.49 (2.88, 6.09)^*^	4.23 (1.85, 6.61)^*^
% single elderly households	0.11 (0.00, 0.22)	-0.01 (-0.09, 0.06)	0.14 (0.04, 0.23)^*^	0.07 (-0.06, 0.20)	-0.25 (-0.46, -0.05)^*^
% detached houses	0.05 (0.02, 0.08)^*^	0.02 (0.00, 0.04)^*^	0.04 (0.01, 0.06)^*^	0.11 (0.08, 0.15)^*^	-0.05 (-0.11, 0.00)^*^
% of people who regularly participated in religious activities	-0.17 (-0.27, -0.08)^*^	0.00 (-0.06, 0.05)	-0.02 (-0.09, 0.05)	-0.22 (-0.33, -0.12)^*^	-0.46 (-0.61, -0.30)^*^
Number of sports facilities per 1,000 persons	-3.93 (-9.28, 1.42)	-2.46 (-6.02, 1.10)	-6.67 (-11.34, -2.01)^*^	-1.52 (-7.97, 4.92)	16.13 (6.33, 25.92)^*^
Park area per person (km^2^)	0.00 (-0.02, 0.01)	-0.01 (-0.03, 0.00)^*^	-0.02 (-0.03, 0.00)^*^	0.00 (-0.02, 0.02)	0.04 (0.01, 0.07)^*^
% current smokers	0.33 (0.15, 0.51)^*^	0.02 (-0.10, 0.14)	0.20 (0.04, 0.36)^*^	0.29 (0.07, 0.51)^*^	0.37 (0.03, 0.71)^*^
% of people exhibiting high-risk drinking	-0.11 (-0.23, 0.02)	0.02 (-0.06, 0.11)	-0.04 (-0.15, 0.07)	-0.08 (-0.23, 0.07)	-0.04 (-0.27, 0.20)
% of population with recognized stress	0.03 (-0.08, 0.13)	-0.01 (-0.08, 0.06)	0.03 (-0.07, 0.12)	0.01 (-0.12, 0.14)	0.12 (-0.08, 0.31)
% of population with obesity	0.11 (-0.04, 0.26)	0.11 (0.01, 0.21)^*^	-0.01 (-0.14, 0.13)	0.10 (-0.08, 0.28)	0.43 (0.15, 0.70)^*^

*Note*) Numbers: Changes in suicide rate per 100,000 persons (95% credible interval)

^*^p < 0.05

## Discussion

In this study, we investigated the association between social environment characteristics and suicide rate in Korea, using nationwide longitudinal data covering all 229 districts over 11 years (2008–2018). We found that 1) poor socioeconomic conditions and isolation characteristics (higher “% of population aged 65 and older eligible for the basic pension”, “% vacant houses in the area”, “% divorce”, and “% detached houses”) were associated with higher suicide rates; 2) higher religious activity and greater access to recreational opportunities and physical activities (accessibility to parks) were associated with lower suicide rates; and 3) higher smoking rates were associated with higher suicide rates. Moreover, these associations with social environment characteristics were found to differ by age group; in general, the associations with socioeconomic status and health behavior characteristics were more pronounced in elderly groups, whereas the association of higher isolation and less recreational opportunities with higher suicide rates was more pronounced in younger age groups.

Lower socioeconomic status has been suggested as a major risk factor of suicide. The suicide rate increased during economic depressions [15, 16], and higher suicide rates among people with lower income and education levels have been reported globally and consistently [1, 11]. This study also showed that poor socioeconomic levels were associated with higher suicide rates at the district-level, as “% of population aged 65 and older eligible for the basic pension” and “% vacant houses in the area” showed a positive association with suicide rate. Moreover, we found that the association between “% of population aged 65 and older eligible for the basic pension” and suicide rate was highest among people aged 60 and older (Table 3). Previous studies also suggested that poverty and economic difficulties are dominant factors in suicide among older persons, along with poor physical health [6]. Thus, this result has crucial implications for suicide prevention policies in Korea, given the distinctively higher suicide rate in the older population than in other age groups.

In addition, the association between suicide rate and % basic pension was more significant in high- and mid-density areas than in low-density areas, although the “% of population aged 65 and older eligible for the basic pension” was highest in low-density areas. We postulate this result is related to larger relative deprivation in mid- and high-density areas; however, this study provided limited epidemiological evidence, and further investigations should be performed to determine the regional differences in relation to poor economic status of the population aged 65 years and older.

Further, social isolation together with lower socioeconomic levels have been identified as another major risk factor for suicide [17–19]. Results from our study were consistent with this finding, as a positive association was found between suicide rate and “% divorce” and “% detached houses”. Moreover, we found that the effects of poor socioeconomic status and social isolation on suicide rate were more prominent in males than in females. We postulate that men’s higher levels of participation in economic activity in Korea might be related to this gender difference. According to the Korea National Statistics Office, the labor force in 2019 comprised 73.5% men and 53.5% women. In addition, these results can be partly explained by the gender difference in social relationship patterns. Previous studies revealed that males are more susceptible to social isolation than females [20, 21] and females usually have larger social networks, receive more social support, and engage more actively in their social relationships than males [22–24]. Although further studies are required, our results suggest the need for gender-differentiated suicide prevention policies that focus on different social vulnerability factors.

This study also found that regional variables related to physical exercise and park availability can lead to a reduction in the suicide rate. Numerous studies reported that increased levels of physical exercise lead to a reduction in stress and depressive disorders that may be related to suicide [25, 26]. Further, although existing results are mixed, a recent systematic review reported a statistically significant negative association between physical activity and suicidal ideation [27]. A Korean study also revealed that more physical activity is associated with less suicidal thoughts and attempts in adolescents [28]. In addition, previous studies have reported that parks and green space provide positive effects, leading to fewer suicidal outcomes (suicide mortality, suicide ideation, and suicide attempts) [29–31], while improving health by encouraging physical and social activities [32, 33]. The effects of physical exercise and park availability on suicide rate in this study were more significant in the youngest age group (aged 10–39) than in other, older, age groups. We consider that this may be associated with outdoor activity patterns in younger people. That is, young people may be more likely than older adults to engage in outdoor and physical activities, and thus the average time spent using parks and sports facilities may also be higher among the former group. Future research is merited to further explore how physical exercise and park use affect suicide in relation to age. Such results could be useful for establishing effective suicide prevention policies for the young generation.

This study also found a positive association between “% current smokers” and suicide rate. Previous studies have addressed smoking as one of the important risk factors for suicide [34] and reported that higher smoking is significantly associated with higher risks of suicidal ideation, planning, and attempts, as well as suicide death [35]. We could not find a positive significant relationship between “% of people exhibiting high-risk drinking” and suicide rate at the district level, and even the male population showed a negative association between high-risk drinking behavior and suicide rate (Table 3). Because numerous existing studies have consistently reported detrimental effects of drinking on suicidal behavior [36, 37], the results of this study should be considered carefully. First, this result might be related to Korean socioeconomic culture. Korean men, especially young males, tend to build social capital primarily in the workplace and through economic activities [38]; a group dinner after work is a major part of the drinking culture of Korea. This implies that there is a possibility that greater alcohol consumption at group dinners can be beneficial to develop social networks and therefore reduce the social isolation that may lead to an increase in suicide risk. Second, the results are estimates at the district level using aggregated data; thus, this association should be examined in greater detail in future studies.

Finally, this study found that the association between social environment characteristics and suicide rate differed by regional urbanicity, with the relationships between social environment characteristics and suicide rate being generally more significant in high-density areas (i.e. more urbanized areas) than other areas (i.e. less urbanized areas). As supported by previous studies, rapid urbanization affects social capital and income inequalities in urban regions, especially among socioeconomically disadvantaged classes, which is closely related to social exclusion [39–41]. We speculate that greater socioeconomic inequalities and social isolation in urban populations might be associated with stronger associations between social environments and suicide rate.

The study had several limitations. First, as mentioned earlier, the study results have a limited interpretation with respect to individual-level associations between social environment characteristics and suicide. Because the mortality data provided by the Korea National Statistics Office does not include individuals’ socioeconomic status and residential addresses, we were unable to examine the specific effects of individual-level socioeconomic status and individual-level environmental exposure data. Therefore, our study results reflected aggregated community-level results. Second, collection of several social environment characteristic variables (“% of people who regularly participated in religious activities”, “% current smokers”, “% of people exhibiting high-risk drinking”, “% of population with recognized stress”, and “% of population with obesity”) was limited to self-report, as these variables were obtained from the Korean Community Health Survey (KCHS) [8]. Although previous studies have reported good quality of self-reported data, and quality control assessments have been performed for KCHS [42], there may be underlying problems in misclassifications and recall bias. Given these possible shortcomings, our study needs to be complemented by data from future individual-level cohort studies.

Nevertheless, our study has some key strengths that can offset its limitations. First, the study analyzed a large nationwide database of suicide deaths in Korea, with more than 154,866 cases over 11 years. By using a large longitudinal data set that covers a relatively long period (11 years), we were able to provide statistically powerful and robust spatiotemporal association between suicide rate and social environmental factors. Moreover, we collected data for a total of 12 annual social environment indicators of regional-level socioeconomic, demographic, urbanicity, general health behaviors, and other environmental characteristics, and analyzed the associations between these annual variables and suicide rates using advanced statistical methods. Finally, by sub-population analyses, we found distinct roles these social environment characteristics perform in reducing or increasing suicide rate measured across densification of areas, sex, and age. These results can be used for establishing evidence-based and targeted suicide prevention policies for each sub-population. To our knowledge, this is the largest study investigating the complex roles of social environment characteristics on suicide rate in Korea.

## Conclusion

This study examined the association between social environment characteristics and suicide rate in Korea, using nationwide longitudinal data from 2008 through 2018. Our study revealed that lower socioeconomic level and greater isolation were associated with a higher suicide rate. Lower religious and physical activities and higher prevalence of smoking were also associated with a higher suicide rate. The associations were generally more significant in more urbanized areas. Our results may provide epidemiological evidence to inform targeted public health interventions for reducing the suicide rate in Korea.

## Supplementary Information

**Additional file 1.**

## Data Availability

Analytic data and R code are available in supplementary files and supplementary materials.

## Abbreviations

OECD: Organisation for Economic Co-operation and Development
WHO: World Health Organization
INLA: INtegrated Laplace Approximations
BYM: Besag-York-Mollie
KCHS: Korean Community Health Survey
