# Supplementary material for: Suicide rate and social environment characteristics in South Korea: the roles of socioeconomic, demographic, urbanicity, general health behaviors, and other environmental factors on suicide rate
Source: BMC Public Health. 2022 Mar 1;22:410. doi: 10.1186/s12889-022-12843-4 (PMC8887086; doi:10.1186/s12889-022-12843-4)
Supplement: Supplementary file 1 — Additional file 1. [file 12889_2022_12843_MOESM1_ESM.docx]

**Supplementary Materials**

**Title.** Suicide rate and social environment characteristics in South Korea: the roles of socioeconomic, demographic, urbanicity, general health behaviors, and other environmental factors on suicide rate.

**Authors.** Hyemin Jang, Whanhee Lee, Yong-ook Kim, and Ho Kim

***Corresponding author**. Ho Kim, PhD. Department of Public Health Science, Graduate School of Public Health, & Institute of Health and Environment, Seoul National University, (Address) 1 Gwanak-ro, Gwanak-gu, Seoul 151-742, Republic of Korea. Telephone: (82) 2 880-2702. E-mail: hokim@snu.ac.kr

**A. Information on Social Environment Indicators**

**1. Socioeconomic status**

**(a) % of population aged 65 and older eligible for the basic pension:** This indicator refers to the percentage of recipients of the basic old-age pension among the older population in each region. The basic old-age pension in South Korea provides monthly financial aids for persons aged 65 or older in the bottom 70% of household income (Pak, 2021). The maximum benefit is at 300,000 won (KRW) per month, which is equivalent to $256 (USD). Thus, we used the indicator in this study to measure the average socioeconomic level of each region.

**(b) % vacant houses in the area:** The indicator denotes the percentage of vacant houses among all the dwellings in each region. Many previous studies (Basset et al., 2006; Kim and Jung, 2017; Roh and Yoo, 2016; Kim et al., 2018), especially in South Korea, have clarified the association between the frequency of vacant houses and the region’s economic level. High unemployment in a region can cause family dissolutions and occurrence of vacant houses (Basset et al., 2006; Kim and Jung, 2017; Roh and Yoo, 2016), and poorer economic level of regions is associated with greater frequency of vacant houses (Mallach, 2006; Roh and Yoo, 2016). Furthermore, studies in South Korea have found that the frequency of abandoned vacant houses in regions is associated with decreased population, the aging of society, deterioration of construction, and density of socially disadvantaged populations (Jeon and Kim, 2016; Park, 2018; Kim et al., 2018). Thus, we used the indicator in this study to measure the average socioeconomic level of each region.

**2. Isolation**

**(a) % divorce:** The indicator, namely the raw divorce rate, refers to the number of regional divorce cases each year divided by the mid-year population. We adopted the concept that divorce is negatively associated with social integration (Glenn and Supanic, 1984; Glenn and Shelton, 1985; White, 1990; Finnas, 1997; Kalmijn and van Groenou, 2005); the term ‘social integration’ is the converse concept of social isolation. Thus, we used the raw divorce rate as an indicator denoting social isolation level.

**(b) % detached houses:** The indicator refers to percentage of detached houses among all dwellings in each region. Previous studies in South Korea have found that social capital levels, such as social support, activity (Lee et al., 2013), and attachment to the neighborhood (Kim, 2014) are lower among residents of detached houses versus apartments. In addition, more than half of single households live within detached houses (Byeon, 2015) in South Korea and residents in detached houses are not surveilled or faced by neighbors to the same extent as residents of other types of dwellings; that is, detached houses are associated with a high level of privacy (Lee et al., 2013). Thus, we postulated this would be suitable as an indicator of regional isolation.

**(c) % single elderly households:** The indicator refers to percentage of older persons living in single households among the regional older population. Social isolation consists of disconnectedness due to loss of social networks (Weiss, 1979); such isolation is related to poor social health conditions, such as being alone, single, rarely engaged in social activity, or lacking social support (Berkman and Syme, 1979; Krause, 1987; Dean et al., 1992; Thoits et al., 2001; Hawkley et al., 2006; Cornwell and Waite, 2009). Moreover, as the environment becomes more urbanized and ages, the social isolation level also increases (Mullins et al., 1996; Vlahov and Galea, 2002). Thus, we used this indicator related to single elderly households to reflect social isolation.

**3. Recreational opportunities/religious and physical activities**

**(a) % people who regularly participated in religious activities:** The indicator refers to the standardized rate of the respondents reporting they participate in the religious activity at least once per month among each regional sample in the survey of community health outcomes and health determinants by the Korean Centers for Disease Control and Prevention. Many previous studies have explained that religion helps expand social networks through religious organizations, events, and belief, and eventually, it enhances the social capital level (Muller and Ellison, 2001; Regnerus, 2003; Yeary et al., 2012). Moreover, due to the increase of social capital, religion can have a positive association with salubrious health outcomes (Hawe and Shiell, 2000; Carson and Chamberlain, 2003; Islam et al., 2006; Yeary et al., 2012). Thus, we adopted the religion indicator to examine if it’s relevant to the measure of suicide.

**(b) Number of sports facilities per 1,000 persons:** The indicator refers to the number of sports facilities per 1,000 residents in each region. Being physically active or playing sports have been studied to have a negative association with suicidal risks such as depression (Paffenbarger Jr et al., 1994), suicidal ideation (Pfledderer et al., 2019; Vancampfort et al., 2018), and hopelessness (Taliaferro et al., 2008). On the other hand, sedentary behaviors, which cause low metabolism, have been denoted to be associated with poor mental health (Rostad et al., 2021; Hoare et al., 2014; Michael et al., 2020). Thus, we postulated the number of facilities in each region would be suitable as an indicator of physical activity.

**(c) Park area per person (km^2^):** The indicator refers to the total geographical size(km^2^) of all the parks per resident in each region. Previous studies have clarified that greater access to parks and the size of parks are positively associated with a high level of physical activity and a decrease in sedentary behaviors (Epstein et al., 2006; Brown et al., 2014). Thus, as in the above items, the number of sports facilities per 1,000 persons, we regarded the park area as one of the indicators of regional physical activity.

**4. Health behavior characteristics**

**(a) % current smokers:** The indicator refers to the standardized rate of respondents who currently smoke. There has been sufficient empirical evidence that smokers are at higher suicide risks such as depression, suicidal ideation, plan, attempt, and death (Pooroljal and Nahid Darvishi, 2016; Hemenway et al., 1993), even though it is not yet clear whether smoking is an indicator of other factors in suicide or whether nicotine itself is a behavioral toxin (Hughes, 2008).

**(b) % of people exhibiting high-risk drinking:** The indicator refers to the standardized rate of respondents who drank more than twice per week over the past year (5+ cans of beer or other equivalent drinks for men; 3+ cans of beer or other equivalent drinks for women). Alcohol consumption has been studied as a factor of suicide risks such as suicidal thoughts, plans, and attempts (Powell et al., 2001; Cherpitel et al., 2004; Glasheen et al., 2015). Moreover, binge drinking, which causes acute intoxication, has been explained to be a greater suicide risk than chronic alcohol use (Cherpitel et al., 2004; Glasheen et al., 2015; Schaffer et al., 2008). Thus, we adopted the high-risk drinking indicator to measure health behavior in this study.

**(c) % of population with recognized stress:** The indicator refers to the standardized rate of respondents who "feel a great deal" or “feel a lot" of stress in daily life. Suicide results from the interaction of more various and complicated psychological, social, socio-psychological, environmental, and genetic factors than other diseases do (Wilcox et al., 2010). However, psychological risks or poor mental health statuses such as depression and stress are well known to be directly related to suicidality (Yang and Clum, 1994; Zeng et al., 2018; Rozanov et al., 2012; Goodin et al., 2019). Thus, we adopted and used the stress indicator for the analysis in our study.

**(d) % of population with obesity:** The indicator refers to the standardized rate of self-reported obesity cases. Researchers have studied the association between obesity and suicidal risks, but the outcomes were not consistent: there have been both positive and negative associations (Carpenter et al., 2000; Heneghan et al., 2012; Klinitzke et al., 2013; Pompili et al., 2006). Obesity and related mental health problems such as anorexia nervosa and bulimia nervosa can be related to suicide risks such as suicidal ideation, attempt, and death (Pompili et al., 2006). Furthermore, especially adolescents and bariatric patients can be greater risk groups for suicide, due to their excessive concern about weight or misconception of aesthetic value (Pompili et al., 2006; Heneghan et al., 2012). However, other studies have represented the inverse relationship and the related biological implication on it: Even though the mechanisms for the association haven’t been clarified enough yet, researchers suggested lower suicide risks with a higher level of BMI could attribute to carbohydrate intake, impaired insulin sensitivity, cerebral serotonin, tryptophan, fatty acid, and cholesterol metabolism (Klinitzke et al., 2013).

**B. Supplementary Tables**

**Table S1. Definition and Survey years of social environment indicators.**

| **Variable name** | **Definition** | **Unit** | **Survey years** | | | | | | | | | | |
| --- | --- | --- | --- | --- | --- | --- | --- | --- | --- | --- | --- | --- | --- |
|  |  |  | **08** | **09** | **10** | **11** | **12** | **13** | **14** | **15** | **16** | **17** | **18** |
| Total suicide rate | Standardized total mortality rate by suicide(X60-X84) per 100,000 persons (standardized based on the 2005 mid-year population) | Person/ 100,000 persons | O | O | O | O | O | O | O | O | O | O | O |
| Male suicide rate | Standardized male mortality rate by suicide(X60-X84) per 100,000 persons (standardized based on the 2005 mid-year population) | Person/ 100,000 persons | O | O | O | O | O | O | O | O | O | O | O |
| Female suicide rate | Standardized female mortality rate by suicide(X60-X84) per 100,000 persons (standardized based on the 2005 mid-year population) | Person/ 100,000 persons | O | O | O | O | O | O | O | O | O | O | O |
| Aged 10~39y suicide rate | Standardized aged 10~39 mortality rate by suicide(X60-X84) per 100,000 persons (standardized based on the 2005 mid-year population) | Person/ 100,000 persons | O | O | O | O | O | O | O | O | O | O | O |
| Aged 40~59y suicide rate | Standardized aged 40~59 mortality rate by suicide(X60-X84) per 100,000 persons (standardized based on the 2005 mid-year population) | Person/ 100,000 persons | O | O | O | O | O | O | O | O | O | O | O |
| Aged 60+y suicide rate | Standardized aged 60 and older mortality rate by suicide(X60-X84) per 100,000 persons (standardized based on the 2005 mid-year population) | Person/ 100,000 persons | O | O | O | O | O | O | O | O | O | O | O |
| % of population aged 65 and older eligible for the basic pension | Percentage of people aged 65 and older who receive basic pensions | % | X | X | X | X | X | X | X | O | O | O | O |
| % vacant houses in the area | (Number of empty houses/Total houses)*100 | % | X | X | O | X | X | X | X | O | O | O | O |
| % divorce | (Number of divorces reported in one year / mid-year population)*1000 *Counting administrative districts based on husband's address | Case/ 1,000 persons | O | O | O | O | O | O | O | O | O | O | O |
| % single elderly households | The proportion of the elderly (65 years of age or older) who live alone among those aged 65 and over. *Ratio of elderly living alone=(number of single-person households aged 65 and over/number of people aged 65 and over)*100 | % | X | X | O | X | X | X | X | O | O | O | O |
| % detached houses | The proportion of detached houses (general, multi-household, detached house with shop) among housings *Detached house : A single-person owned house that cannot be owned or sold separately even if it is designed for multiple households | % | X | X | O | X | X | X | X | O | O | O | O |
| % of people who regularly participated in religious activities | Persons who regularly participate in social activities (religious activities) at least once a month/respondents surveyed*100 *Age-standardized based on the 2005 estimated population | % | X | X | X | O | X | O | X | O | X | O | X |
| Number of sports facilities per 1,000 persons | Number of sports facility/resident registered population*1000 | Unit/ 1,000 persons | O | O | O | O | O | O | O | O | O | O | O |
| Park area per person (km^2^) | Park area per person = Park area/ total number of people | m^2^/ person | O | O | O | O | O | O | O | O | O | O | O |
| % current smokers | Percentage of people who have smoked more than 5 packs (100 cigarettes) in their lifetime who currently smoke ("every day" or "sometimes") *Age-standardized based on the 2005 estimated population | % | O | O | O | O | O | O | O | O | O | O | O |
| % of people exhibiting high-risk drinking | Percentage of people who drank more than twice a week over the past year (more than 7 glasses (or 5 cans of beer) for men and more than 5 glasses (or 3 cans of beer) for women) *Age-standardized based on the 2005 estimated population | % | O | O | O | O | O | O | O | O | O | O | O |
| % of population with recognized stress | Persons who responds to "feel a great deal" or "feel a lot" of stress in daily life / Respondents surveyed*100 *Age-standardized based on the 2005 estimated population | % | O | O | O | O | O | O | O | O | O | O | O |
| % of population with obesity | Persons with a body mass index(kg/m2) of 25 or higher / Respondents surveyed*100 (self-report) *Age-standardized based on the 2005 estimated population | % | O | O | O | O | O | O | O | O | O | O | O |

**Table S2. Correlations among social environment indicators.**

|  | % of population aged 65 and older eligible for the basic pension | % vacant houses in the area | % divorce | % single elderly households | % detached houses | % of people who regularly participated in religious activities | Number of sports facilities per 1,000 persons | Park area per person (km^2^) | % current smokers | % of people exhibiting high-risk drinking | % of population with recognized stress | % of population with obesity |
| --- | --- | --- | --- | --- | --- | --- | --- | --- | --- | --- | --- | --- |
| % of population aged 65 and older eligible for the basic pension | 1.00 |  |  |  |  |  |  |  |  |  |  |  |
| % vacant houses in the area | 0.61 | 1.00 |  |  |  |  |  |  |  |  |  |  |
| % divorce | 0.01 | -0.09 | 1.00 |  |  |  |  |  |  |  |  |  |
| % single elderly households | 0.72 | 0.52 | -0.15 | 1.00 |  |  |  |  |  |  |  |  |
| % detached houses | 0.75 | 0.71 | -0.19 | 0.69 | 1.00 |  |  |  |  |  |  |  |
| % of people who regularly participated in religious activities | -0.42 | -0.36 | 0.02 | -0.37 | -0.32 | 1.00 |  |  |  |  |  |  |
| Number of sports facilities per 1,000 persons | 0.27 | 0.35 | -0.02 | 0.20 | 0.36 | -0.15 | 1.00 |  |  |  |  |  |
| Park area per person (km^2^) | 0.11 | 0.23 | -0.07 | 0.15 | 0.14 | -0.07 | 0.08 | 1.00 |  |  |  |  |
| % current smokers | 0.17 | -0.01 | 0.42 | 0.08 | 0.09 | -0.13 | 0.03 | -0.05 | 1.00 |  |  |  |
| % of people exhibiting high-risk drinking | 0.05 | 0.13 | 0.25 | -0.07 | 0.00 | -0.20 | 0.08 | -0.08 | 0.17 | 1.00 |  |  |
| % of population with recognized stress | -0.40 | -0.34 | 0.21 | -0.39 | -0.43 | 0.22 | -0.14 | -0.16 | 0.10 | 0.19 | 1.00 |  |
| % of population with obesity | 0.08 | 0.24 | 0.06 | -0.03 | 0.08 | -0.21 | 0.10 | -0.05 | -0.21 | 0.37 | 0.04 | 1.00 |

**Table S3. Sensitivity Analysis.** Association between social environment characteristics and suicide rate in the total population

|  | **Total population** | **Without “% of population aged 65 and older eligible for the basic pension”** | **Without “% detached houses”** | **Without both variables** |
| --- | --- | --- | --- | --- |
| % population aged 65 and older eligible for the basic pension | 0.09 (0.04, 0.14)^*^ | - | 0.14 (0.09, 0.18)^*^ | - |
| % vacant houses in the area | 0.16 (0.07, 0.25)^*^ | 0.16 (0.07, 0.26)^*^ | 0.25 (0.16, 0.33)^*^ | 0.31 (0.22, 0.39)^*^ |
| % divorce | 1.88 (0.99, 2.76)^*^ | 2.08 (1.21, 2.96)^*^ | 1.41 (0.53, 2.28)^*^ | 1.54 (0.64, 2.42)^*^ |
| % single elderly households | 0.05 (-0.02, 0.12) | 0.09 (0.02, 0.15)^*^ | 0.08 (0.01, 0.15)^*^ | 0.16 (0.10, 0.23)^*^ |
| % detached houses | 0.04 (0.02, 0.06)^*^ | 0.06 (0.04, 0.08)^*^ | - | - |
| % people who regularly participated in religious activities | -0.11 (-0.17, -0.05)^*^ | -0.13 (-0.19, -0.07)^*^ | -0.11 (-0.17, -0.05)^*^ | -0.14 (-0.20, -0.08)^*^ |
| Number of sports facilities per 1,000 persons | -2.85 (-6.23, 0.52) | -2.72 (-6.10, 0.66) | -1.81 (-5.19, 1.56) | -0.90 (-4.30, 2.49) |
| Park area per person (km^2^) | -0.01 (-0.02, 0.00) | -0.01 (-0.02, 0.00) | -0.01 (-0.02, 0.00) | -0.01 (-0.03, 0.00)^*^ |
| % current smokers | 0.17 (0.06, 0.28)^*^ | 0.18 (0.07, 0.30)^*^ | 0.18 (0.06, 0.29)^*^ | 0.20 (0.09, 0.32)^*^ |
| % people exhibiting a high risk drinking | -0.05 (-0.13, 0.03) | -0.04 (-0.12, 0.03) | -0.05 (-0.13, 0.03) | -0.04 (-0.12, 0.04) |
| % population with recognized stress | -0.01 (-0.07, 0.06) | -0.02 (-0.08, 0.05) | -0.03 (-0.09, 0.04) | -0.06 (-0.12, 0.00) |
| % population that is obese | 0.09 (0.00, 0.18) | 0.10 (0.00, 0.19)^*^ | 0.10 (0.00, 0.19)^*^ | 0.11 (0.02, 0.21)^*^ |
| Note) Changes in suicide rate per 100,000 (95% confidence interval) | | | | |
| *p<0.05 | | | | |

**C. Supplementary Figures**

**Figure S1.** Geographical distribution of the average of each social environment characteristics across 229 districts in Korea during the period 2008–2018

| % of population aged 65 and older eligible for the basic pension  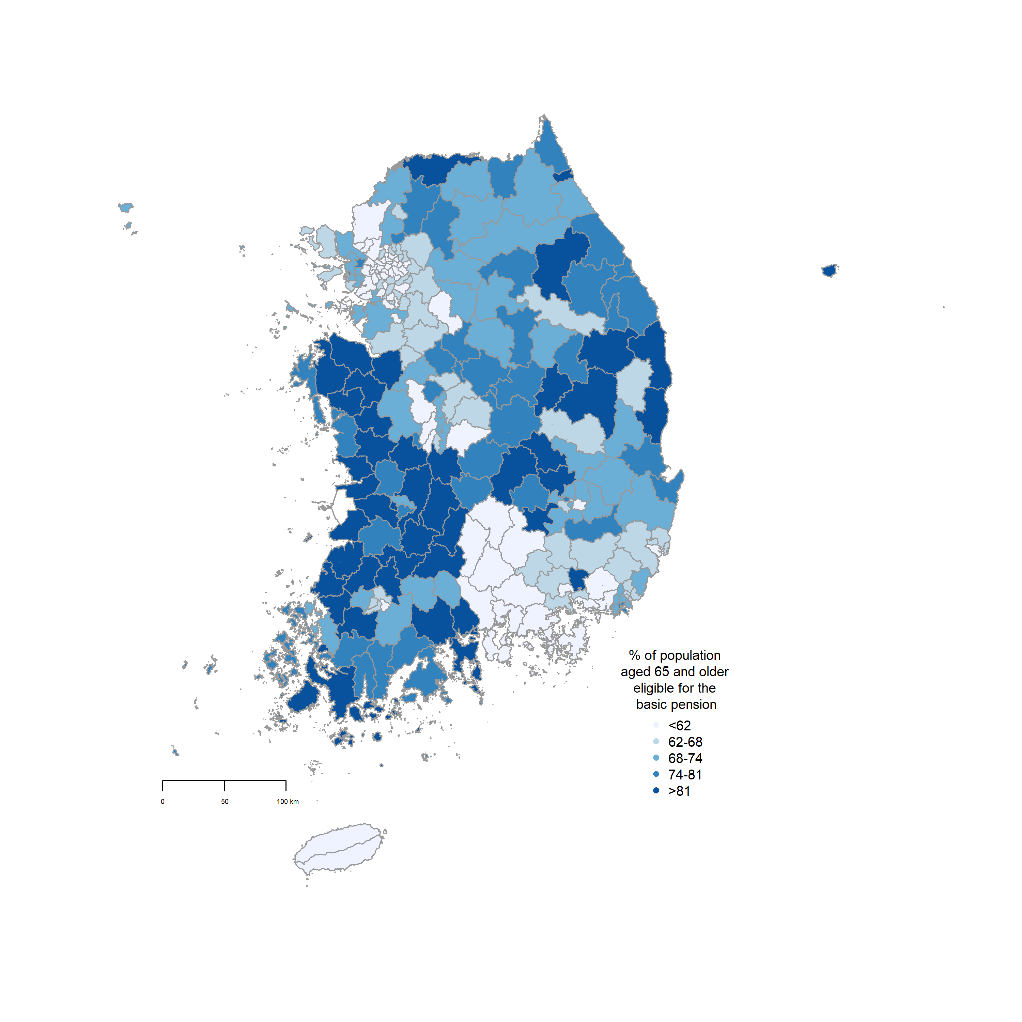 | % vacant houses in the area  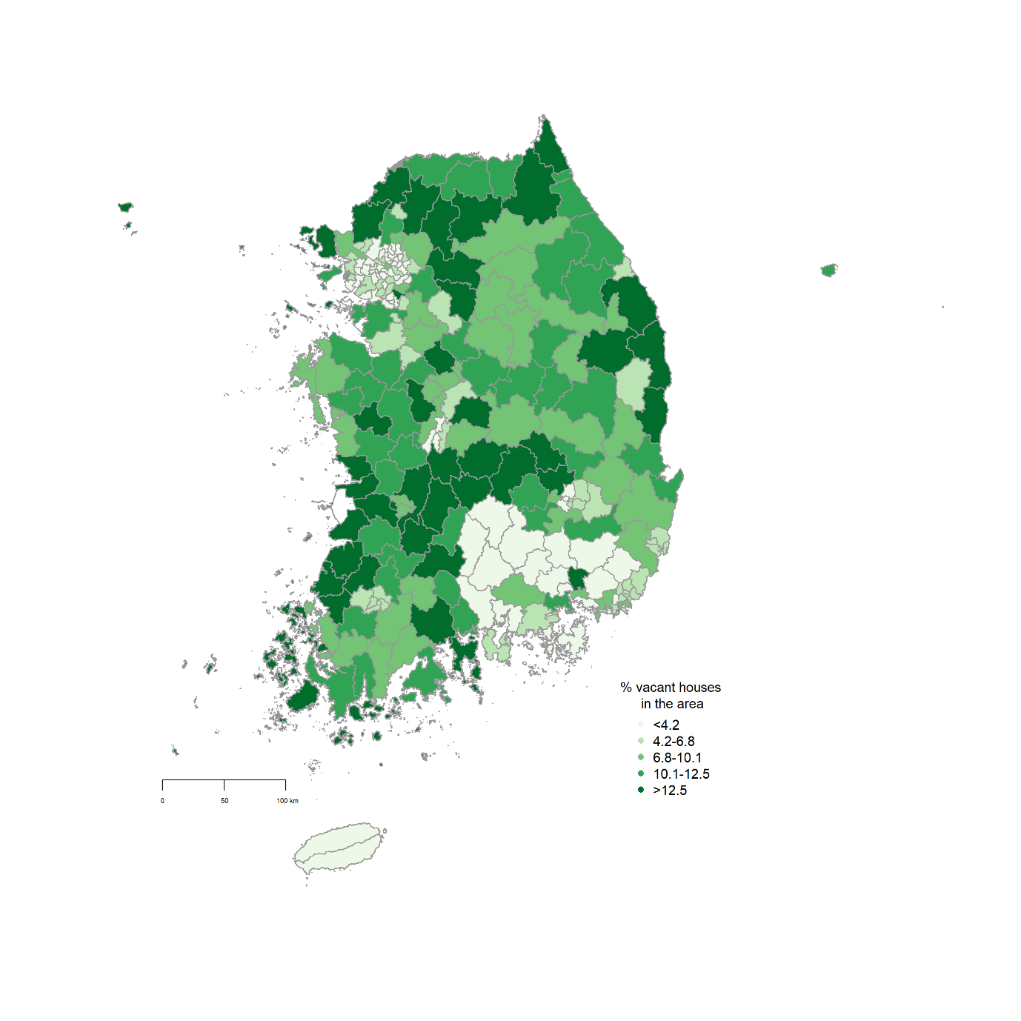 |
| --- | --- |

| % divorce 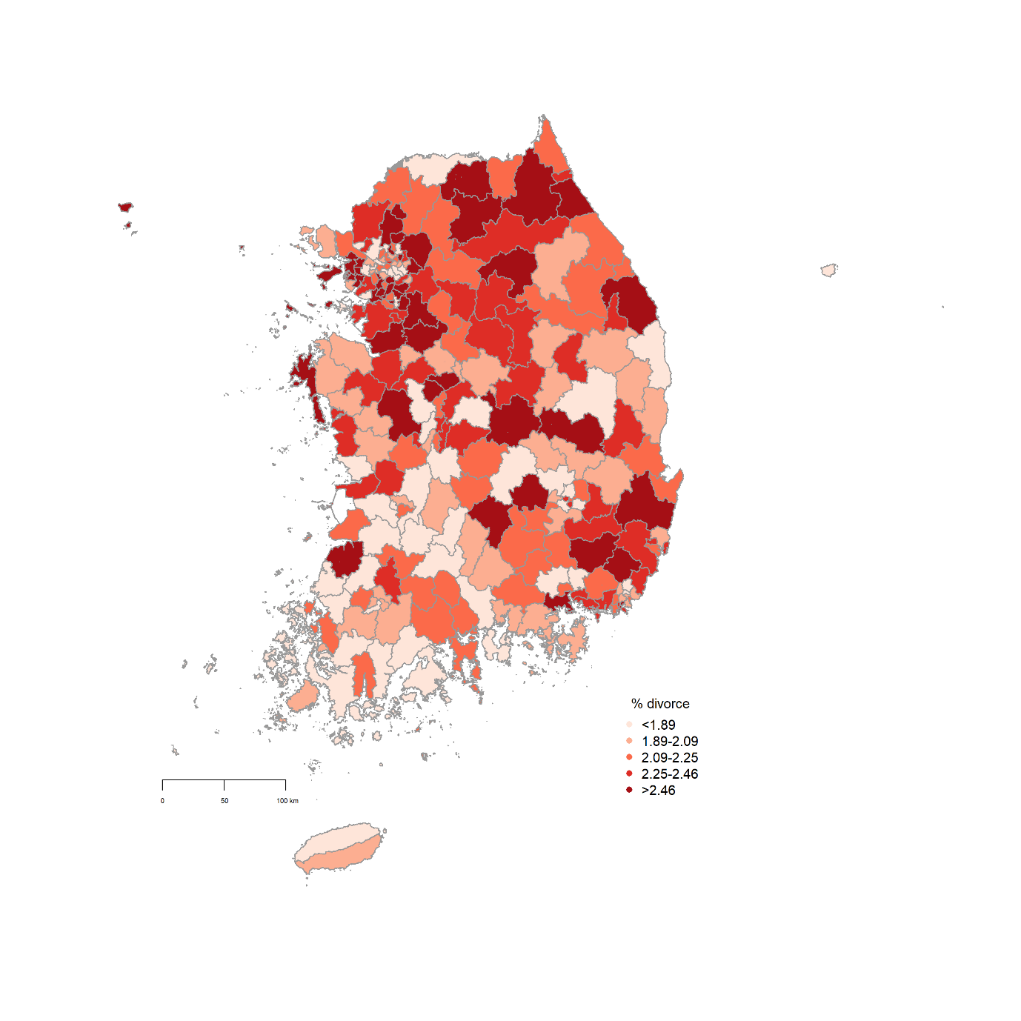 | % single elderly households  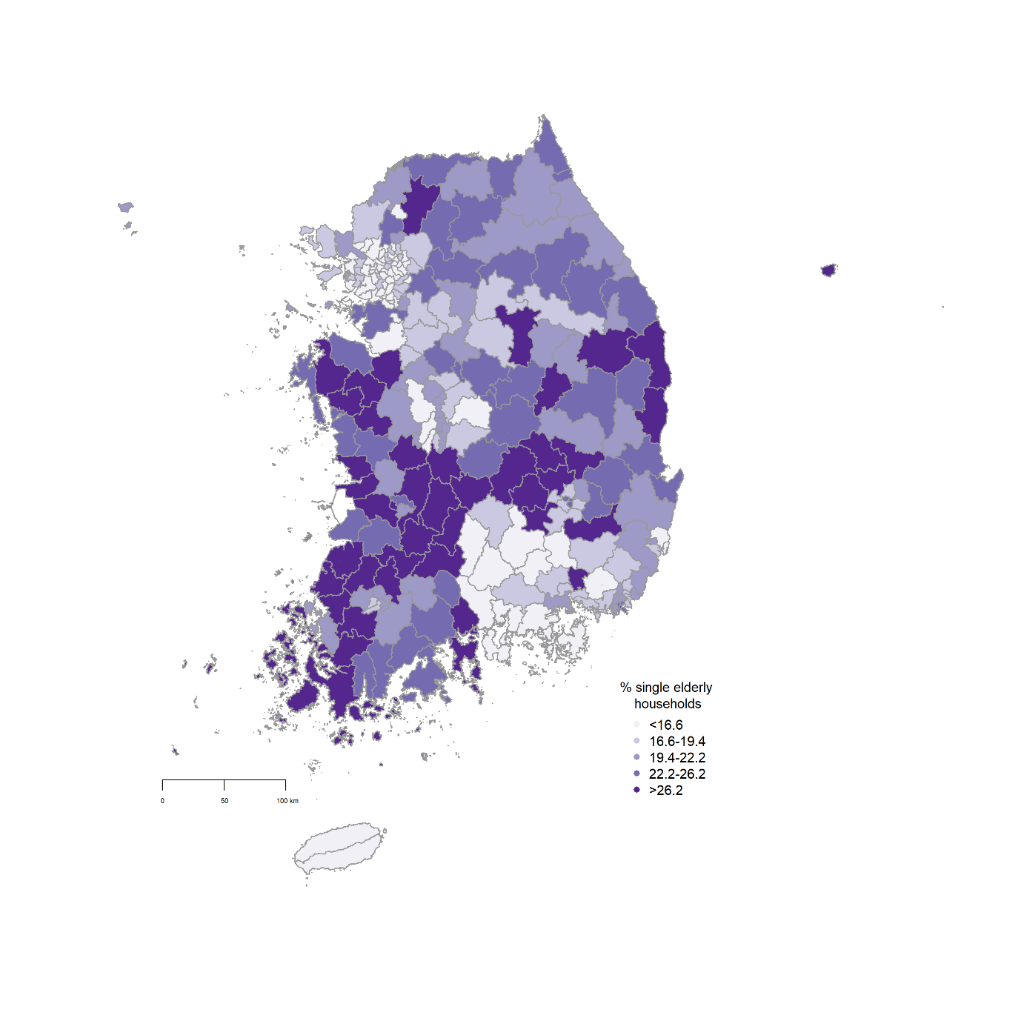 |
| --- | --- |

| % detached houses  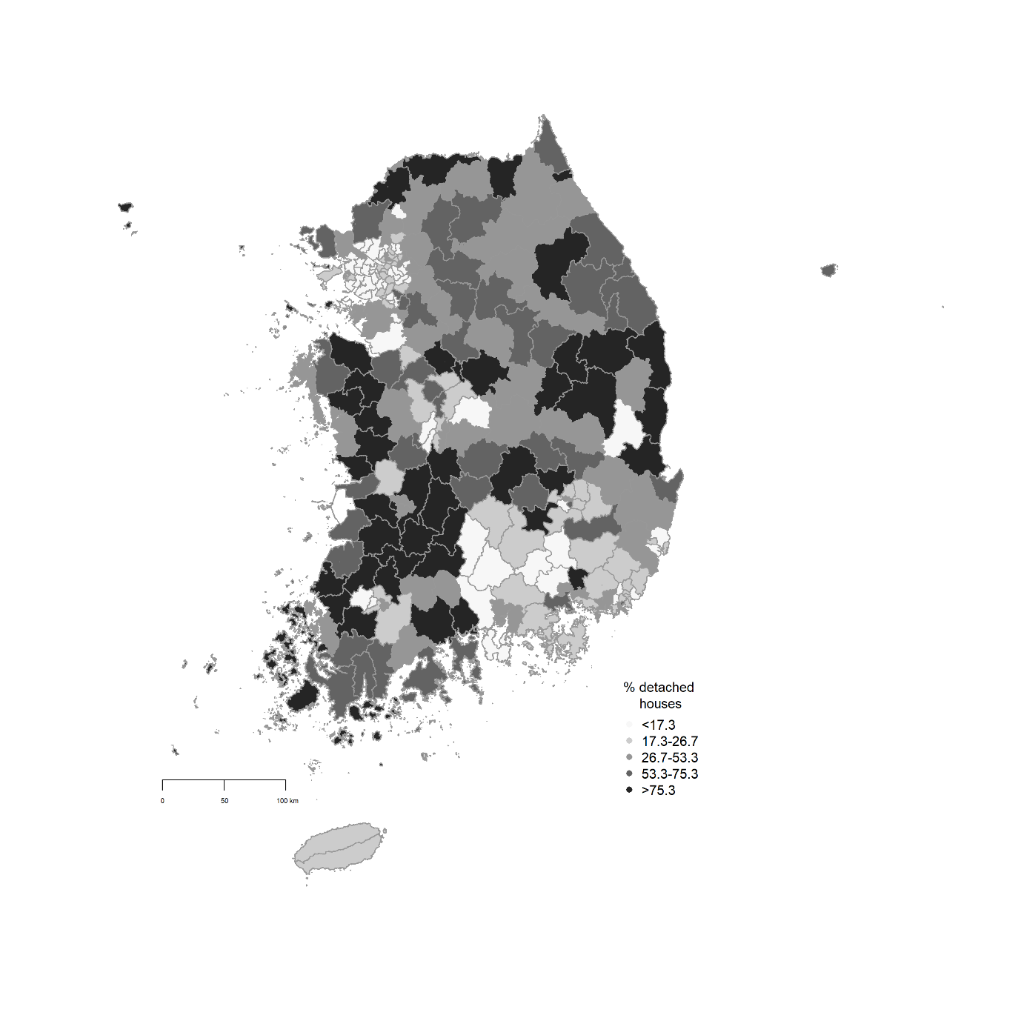 | % of people who regularly participated in religious activities  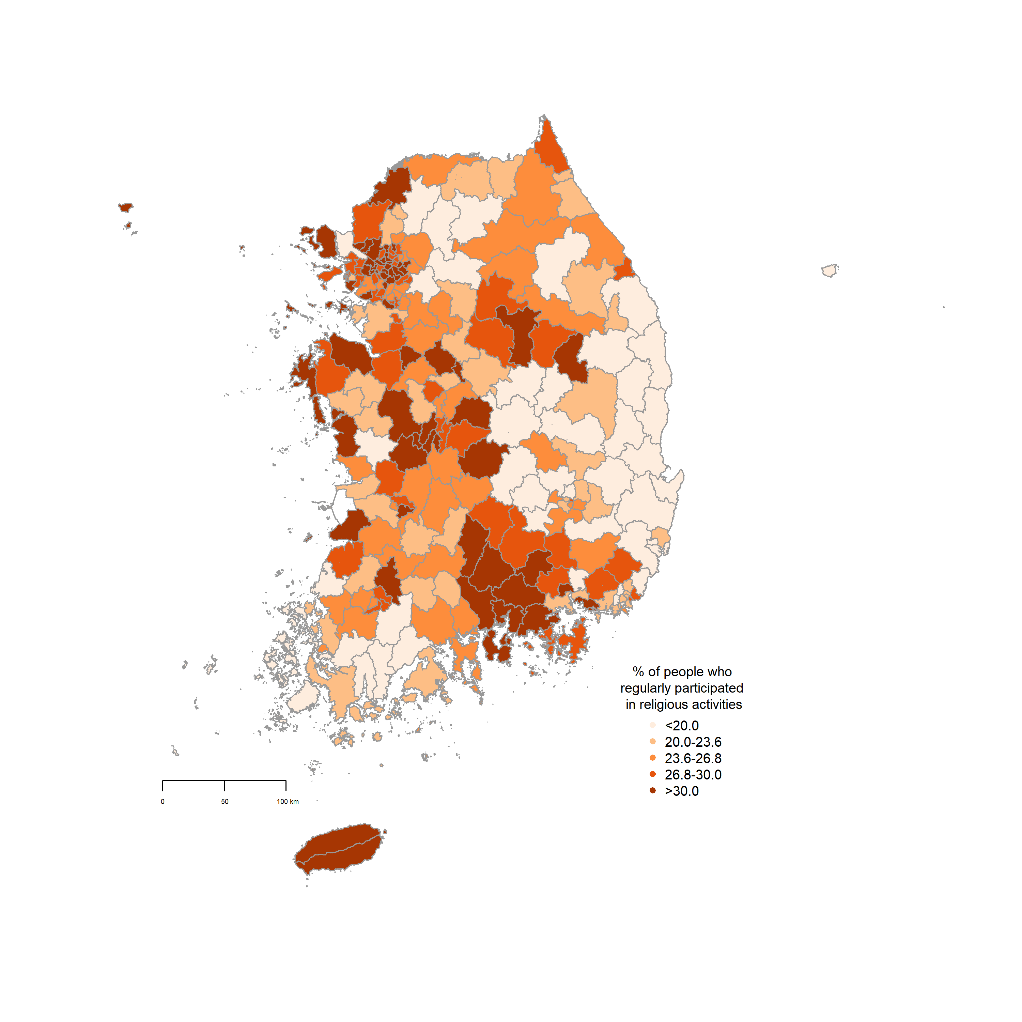 |
| --- | --- |

| Number of sports facilities per 1,000 persons  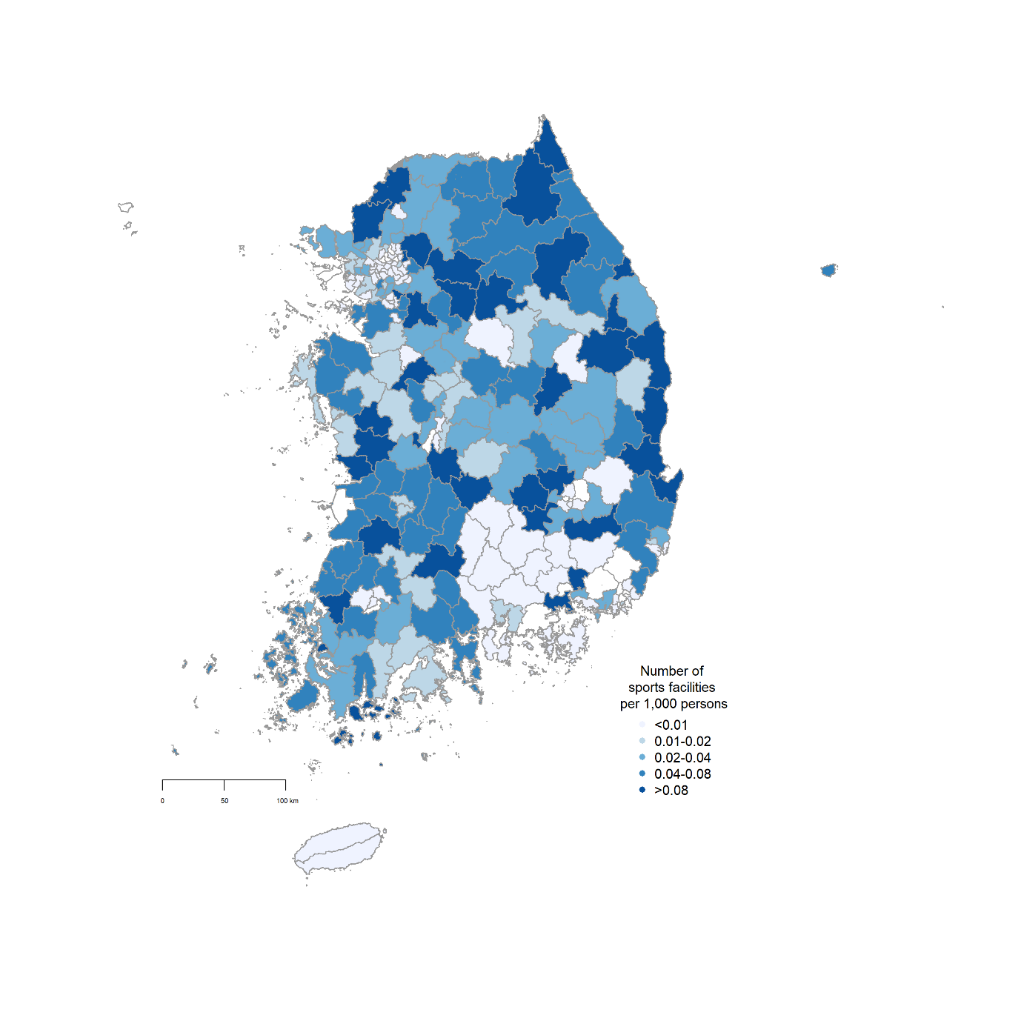 | Park area per person (km^2^)  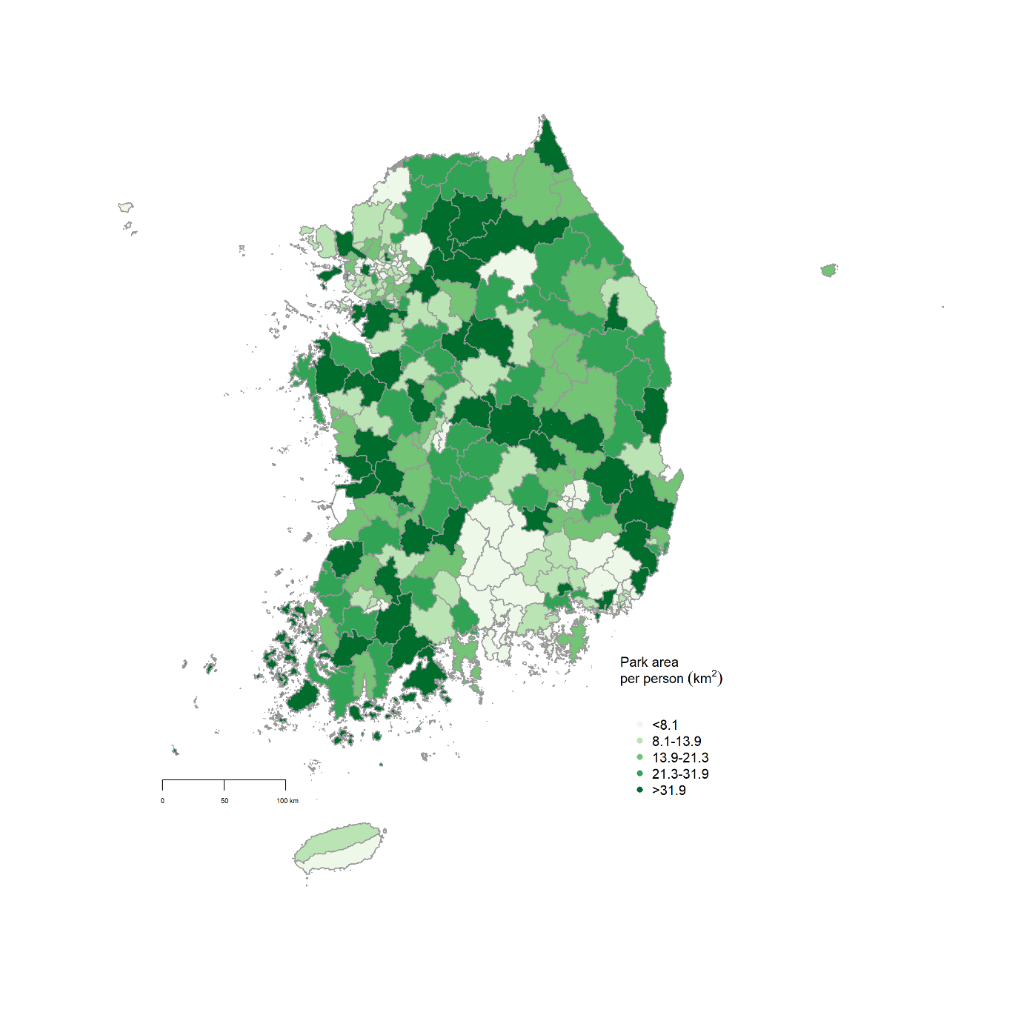 |
| --- | --- |

| % current smokers  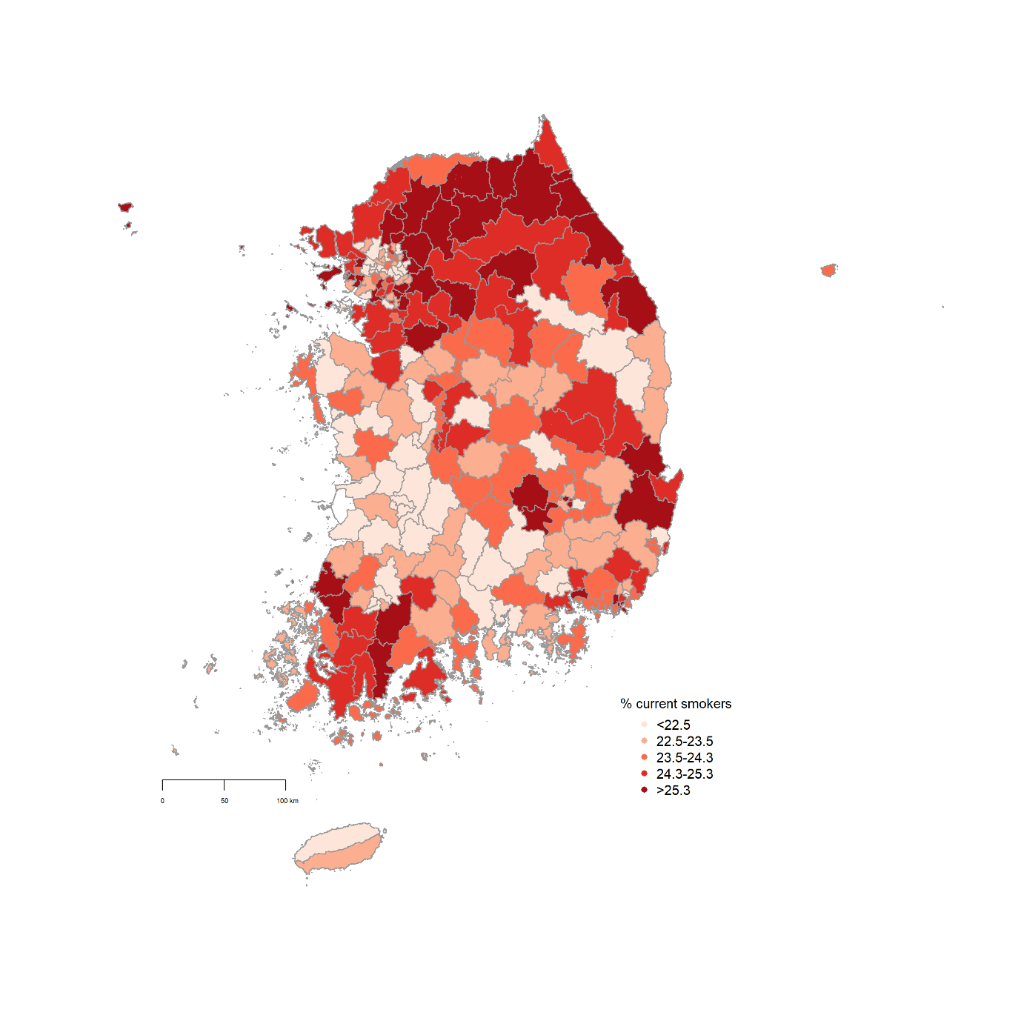 | % of people exhibiting high-risk drinking  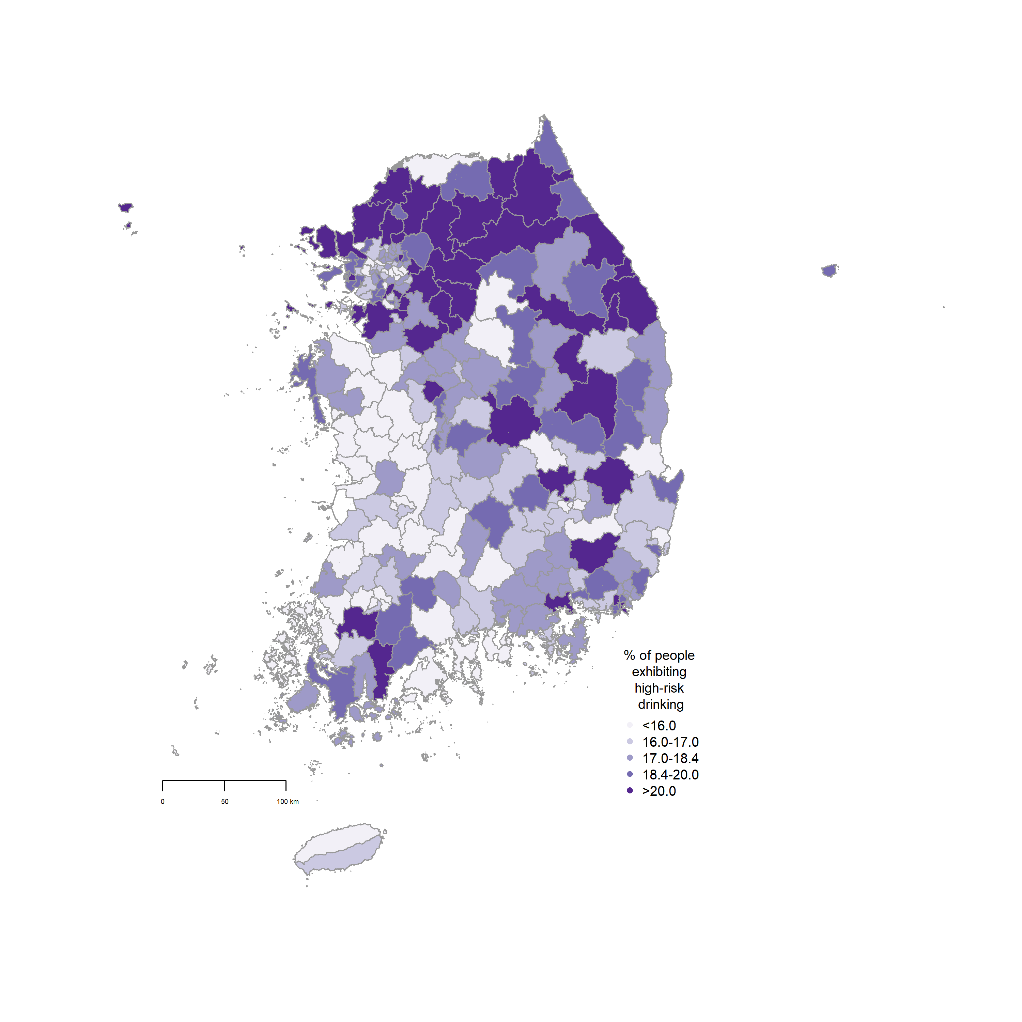 |
| --- | --- |

| % of population with recognized stress  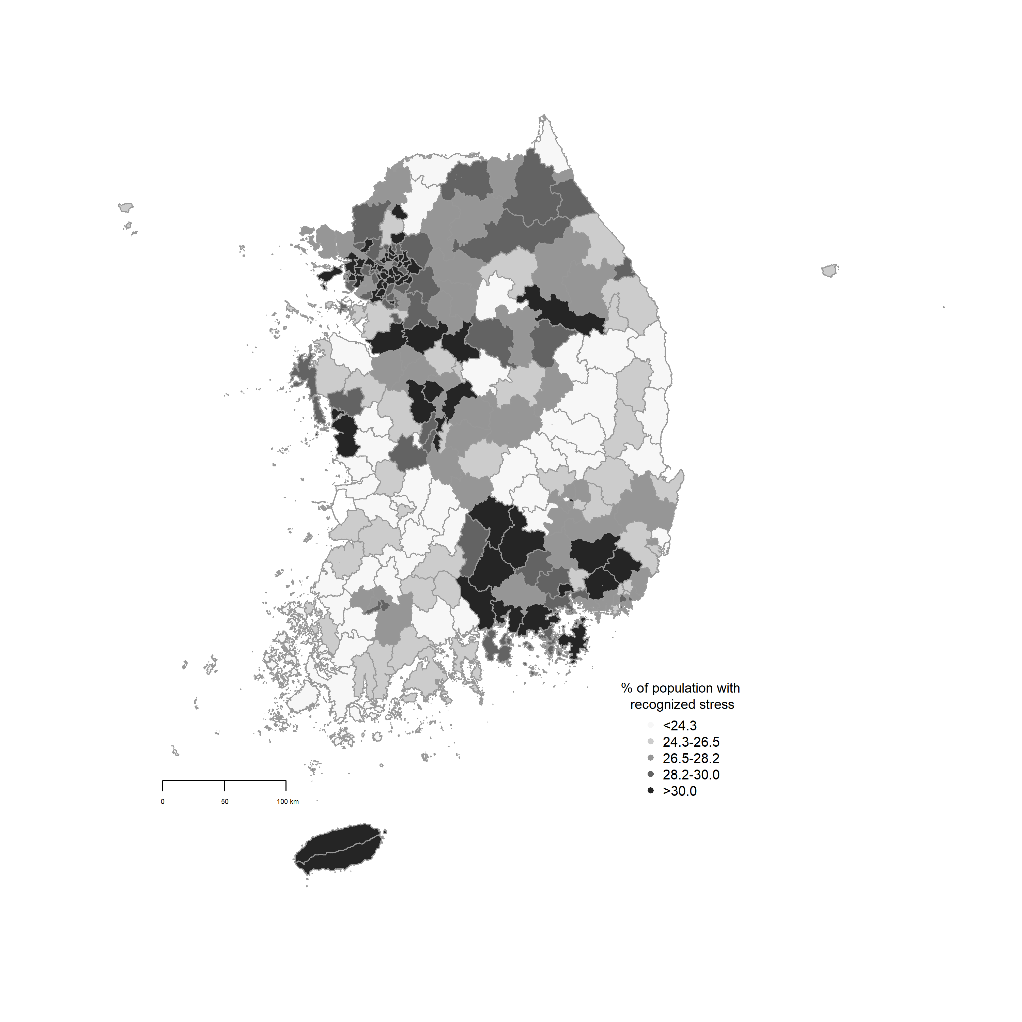 | % of population with obesity  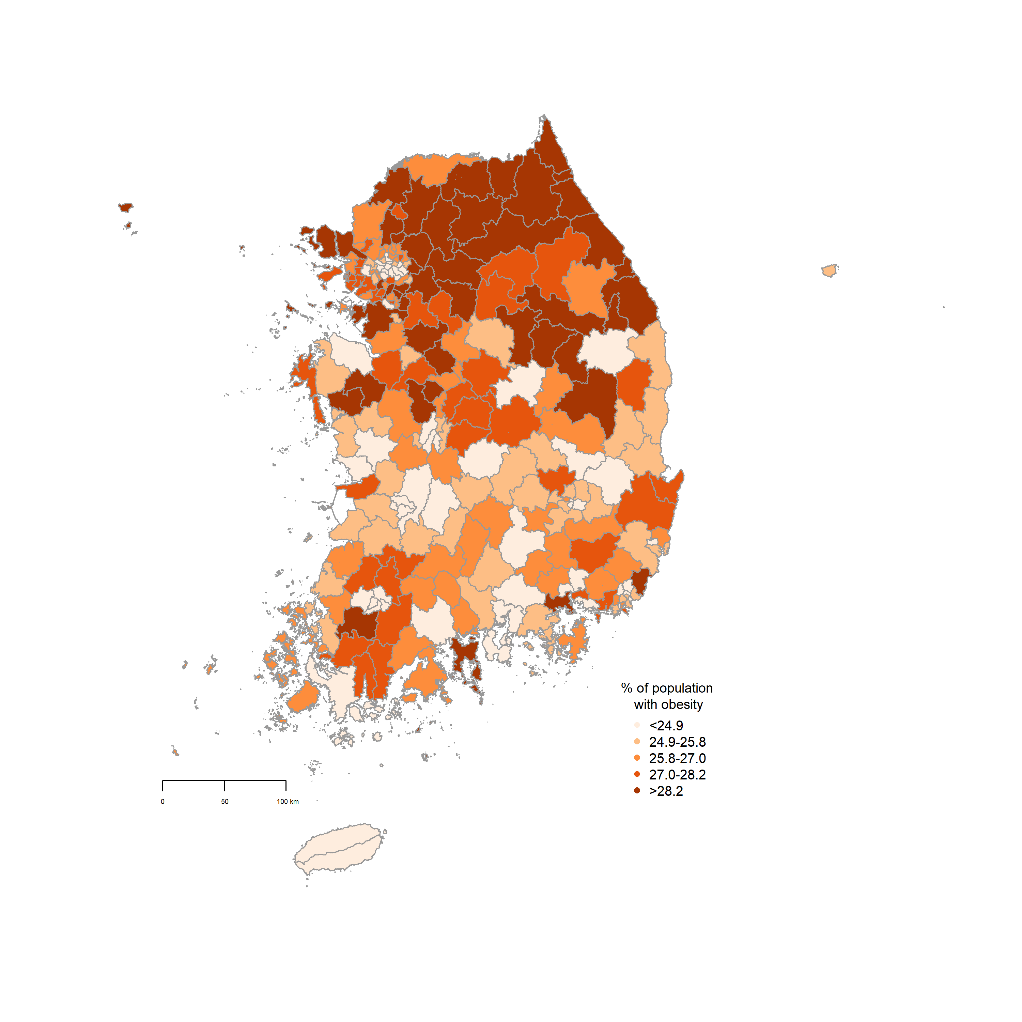 |
| --- | --- |

**D. Statistical Analysis Code**

### INLA ###

library(INLA)

library(maptools)

library(spdep)

data0818<-read.csv(“data0818.csv”, header=T)

data_gen <- readShapePoly("sigungu2009.shp")

gen_temp <- poly2nb(data_gen)

nb2INLA("korea.graph", gen_temp)

KOR.adj <- paste(getwd(), "/korea.graph",sep="")

data0818$chs_no2 <- data0818$chs_no3 <- data0818$chs_no

formula <- suiall ~ 1 + bspension + vacant + divorce + solioldratio + res_detach + religion + gymnum_k + pkarea_i + smkall + y_hdrink + stress + obe + factor(year) + lat + long + f(chs_no2, model="iid") + f(chs_no3, model="bym", graph=KOR.adj)

model <- inla(formula, family="gaussian", data=data0818)

**Supplementary References**

Pak TY: **What are the effects of expanding social pension on health? Evidence from the Basic Pension in South Korea**. *The Journal of the Economics of Ageing* 2021, **18**:100287.

Bassett EM, Schweitzer J, Panken SJLIoLPWP: **Understanding housing abandonment and owner decision-making in Flint, Michigan: An exploratory analysis**. *Lincoln Institute of Land Policy Working Paper* 2006:1-62.

Berkman LF, Syme SL: **Social networks, host resistance, and mortality: a nine-year follow-up study of Alameda County residents**. *American journal of Epidemiology* 1979, **109**(2):186-204.

Brown G, Schebella MF, Weber DJL, planning u: **Using participatory GIS to measure physical activity and urban park benefits**. *Landscape and urban planning* 2014, **121**:34-44.

Byeon M: **Single Person Household and Urban Policy in Seoul**. *Korean Journal of Culture and Social Issues* 2015, **21**(3):551-573.

Carlson ED, Chamberlain RM: **Social capital, health, and health disparities**. *Journal of Nursing Scholarship* 2003, **35**(4):325-331.

Carpenter KM, Hasin DS, Allison DB, Faith MS: **Relationships between obesity and DSM-IV major depressive disorder, suicide ideation, and suicide attempts: results from a general population study**. *American Journal of Public Health* 2000, **90**(2):251.

Cherpitel CJ, Borges GL, Wilcox HC: **Acute alcohol use and suicidal behavior: a review of the literature**. *Alcoholism: clinical experimental research* 2004, **28**:18S-28S.

Cornwell EY, Waite LJ: **Social disconnectedness, perceived isolation, and health among older adults**. *Journal of health and social behavior* 2009, **50**(1):31-48.

Epstein LH, Raja S, Gold SS, Paluch RA, Pak Y, Roemmich J: **Reducing sedentary behavior: the relationship between park area and the physical activity of youth**. *Journal of Psychological Science* 2006, **17**(8):654-659.

Finnäs F: **Social integration, heterogeneity, and divorce: The case of the Swedish-speaking population in Finland**. *Acta Sociologica* 1997, **40**(3):263-277.

Glasheen C, Pemberton MR, Lipari R, Copello EA, Mattson ME: **Binge drinking and the risk of suicidal thoughts, plans, and attempts**. *Addictive Behaviors* 2015, **43**:42-49.

Glenn ND, Shelton BA: **Regional differences in divorce in the United States**. *Journal of Marriage and the Family* 1985:641-652.

Glenn ND, Supancic M: **The social and demographic correlates of divorce and separation in the United States: An update and reconsideration**. *Journal of Marriage and the Family* 1984:563-575.

Goodin CA, Prendergast DM, Pruitt LD, Smolenski DJ, Wilson NY, Skopp N, Hoyt T: **Financial hardship and risk of suicide among US Army personnel**. *Psychological Services* 2019, **16**(2):286.

Hawe P, Shiell A: **Social capital and health promotion: a review**. *Journal of Social science and Medicine* 2000, **51**(6):871-885.

Hawkley LC, Masi CM, Berry JD, Cacioppo JT: **Loneliness is a unique predictor of age-related differences in systolic blood pressure**. *Psychology and aging* 2006, **21**(1):152.

Heneghan HM, Heinberg L, Windover A, Rogula T, Schauer PR, Diseases R: **Weighing the evidence for an association between obesity and suicide risk**. *Surgery for Obesity* 2012, **8**(1):98-107.

Hoare E, Skouteris H, Fuller-Tyszkiewicz M, Millar L, Allender S: **Associations between obesogenic risk factors and depression among adolescents: a systematic review**. *Obes Rev* 2014, **15**(1):40-51.

Hughes JR: **Smoking and suicide: a brief overview**. *Alcohol and Drug Dependence* 2008, **98**(3):169-178.

Islam MK, Merlo J, Kawachi I, Lindström M, Gerdtham U-GJIjfeih: **Social capital and health: Does egalitarianism matter? A literature review**. *International journal for equity in health* 2006, **5**(1):1-28.

Jeon Y-m, Kim S-h: **The Causes and Characterstics of Housing Abandonment in an Inner-city Neighborhood - Focused on the Sungui-dong Area, Nam-gu, Incheon**. *Journal of The Urban Design Insitute of Korea* 2016, **17**(1):83-100.

Kalmijn M, van Groenou MB: **Differential effects of divorce on social integration**. *Journal of Social Personal Relationships* 2005, **22**(4):455-476.

Kim H-j, Han H-g, Yuh K-h: **Effects of Regional Characteristics on Housing Abandonment: Focused on Demographic Change and Residential Environment**. *Korean Journal of Urban Studies* 2018(13):43-76.

Kim H-j, Lee J-g, Jung I: **Forecasting for Spatial Patterns of Empty Houses in Rural Area Using Probabilistic Choice Model**. *Journal of The Residential Environment Institute of Korea* 2016, **14**(3):15-27.

Kim Y-m: **A Study on Residential Influential Factors on Community Social Capital in Daejeon Metropolitan City**. *Daejeon University* 2014.

Klinitzke G, Steinig J, Blüher M, Kersting A, Wagner B: **Obesity and suicide risk in adults - a systematic review**. *Journal of Affective Disorders* 2013, **145**(3):277-284.

Krause N: **Satisfaction with social support and self-rated health in older adults**. *The Gerontologist* 1987, **27**(3):301-308.

Lee Y-s, Yoon G-y, Jang J-w, Ahn C-h: **A Study on Social Relationship of Housing Types for Social Health of the Elderly**. *KOREAN INSTITUTE OF INTERIOR DESIGN JOURNAL* 2013, **22**(5):258-266.

Mallach A: **Bringing buildings back: From abandoned properties to community assets: A guidebook for policymakers and practitioners**. *Rutgers University Press* 2006.

Michael SL, Lowry R, Merlo C, Cooper AC, Hyde ET, McKeon RJPmr: **Physical activity, sedentary, and dietary behaviors associated with indicators of mental health and suicide risk**. *Preventive medicine reports* 2020, **19**:101153.

Muller C, Ellison CGJSF: **Religious involvement, social capital, and adolescents' academic progress: Evidence from the National Education Longitudinal Study of 1988**. *Sociological Focus* 2001, **34**(2):155-183.

Mullins LC, Elston CH, Gutkowski SM: **Social determinants of loneliness among older Americans**. *Genetic, social, and general psychology monographs* 1996.

Paffenbarger Jr R, Lee IM, Leung R: **Physical activity and personal characteristics associated with depression and suicide in American college men**. *Acta Psychiatrica Scandinavica* 1994, **89**:16-22

Pak T-Y: **What are the effects of expanding social pension on health? Evidence from the Basic Pension in South Korea**. *The Journal of the Economics of Ageing* 2021, **18**:100287.

Park S-N: **The Local Characteristics of Empty Homes in Deprived Areas and Implications for Revitalizing EmptyHomes - Focusing the Perception of Residents and Local Coordinators** *Journal of The Urban Design Insitute of Korea* 2018, **19**(1):5-20.

Pfledderer CD, Burns RD, Brusseau TA: **School environment, physical activity, and sleep as predictors of suicidal ideation in adolescents: Evidence from a national survey**. *Journal of Adolescence* 2019, **74**:83-90.

Powell KE, Kresnow M-j, Mercy JA, Potter LB, Swann AC, Frankowski RF, Lee RK, Bayer TL: **Alcohol consumption and nearly lethal suicide attempts**. *Suicide and Life-Threatening Behavior* 2001, **32**(Supplement to Issue 1):30-41.

Regnerus MDJRorr: **Religion and positive adolescent outcomes: A review of research and theory**. *Review of religious research* 2003:394-413.

Roh M, Yoo S: **A Study on the Cause of Abandoned Vacant Houses**. *Korea Real Estate Review* 2016, **26**:7-21.

Rostad WL, Basile KC, Clayton HB: **Association Among Television and Computer/Video Game Use, Victimization, and Suicide Risk Among U.S. High School Students**. *J Interpers Violence* 2021, **36**(5-6):2282-2305.

Rozanov V, Carli V, health p: **Suicide among war veterans**. *International Journal of Environmental Research* 2012, **9**(7):2504-2519.

Schaffer M, Jeglic EL, Stanley B: **The relationship between suicidal behavior, ideation, and binge drinking among college students**. *Archives of Suicide Research* 2008, **12**(2):124-132.

Taliaferro LA, Rienzo BA, Miller MD, Pigg Jr RM, Dodd VJ: **High school youth and suicide risk: exploring protection afforded through physical activity and sport participation**. *Journal of School Health* 2008, **78**(10):545-553.

Thoits PA, Hewitt LN: **Volunteer work and well-being**. *Journal of health and social behavior* 2001:115-131.

Vancampfort D, Hallgren M, Firth J, Rosenbaum S, Schuch FB, Mugisha J, Probst M, Van Damme T, Carvalho AF, Stubbs B: **Physical activity and suicidal ideation: A systematic review and meta-analysis**. *Journal of Affective Disorders* 2018, **225**:438-448.

Vlahov D, Galea S: **Urbanization, urbanicity, and health**. *Journal of Urban Health* 2002, **79**(1):S1-S12.

Weiss RS: **Loneliness: The experience of emotional and social isolation**. *The MIT Press* 1973.

Wilcox HC, Arria AM, Caldeira KM, Vincent KB, Pinchevsky GM, O'Grady KE: **Prevalence and predictors of persistent suicide ideation, plans, and attempts during college**. *Journal of Affective Disorders* 2010, **127**(1-3):287-294.

White L.K.: **Determinants of divorce: A review of research in the eighties**. *Journal of Marriage and the Family* 1990:904-912.

Yang B, Clum GA: **Life stress, social support, and problem‐solving skills predictive of depressive symptoms, hopelessness, and suicide ideation in an Asian student population: A test of a model**. *Suicide and Life-Threatening Behavior* 1994, **24**(2):127-139.

Zeng B, Zhao J, Zou L, Yang X, Zhang X, Wang W, Zhao J, Chen J: **Depressive symptoms, post-traumatic stress symptoms and suicide risk among graduate students: The mediating influence of emotional regulatory self-efficacy**. *Psychiatry Research* 2018, **264**:224-230.

Hemenway, D., Solnick, S. J., & Colditz, G. A. **Smoking and suicide among nurses**. *American Journal of Public Health* 1993, 83(2), 249-251.

Yeary, K. H. C. K., Ounpraseuth, S., Moore, P., Bursac, Z., & Greene, P. **Religion, social capital, and health**. *Review of Religious Research* 2012, 54(3), 331-347.

Poorolajal, J., & Darvishi, N. **Smoking and suicide: a meta-analysis**. *PloS one* 2016, 11(7), e0156348.

Pompili, M., Girardi, P., Tatarelli, G., Ruberto, A., & Tatarelli, R. **Suicide and attempted suicide in eating disorders, obesity and weight–image concern**. *Eating behaviors* 2006, 7(4), 384-394.
